# Modeling Cancer Progression via Pathway Dependencies

**DOI:** 10.1371/journal.pcbi.0040028

**Published:** 2008-02-15

**Authors:** Elena J Edelman, Justin Guinney, Jen-Tsan Chi, Phillip G Febbo, Sayan Mukherjee

**Affiliations:** 1 Institute for Genome Sciences and Policy, Duke University, Durham, North Carolina, United States of America; 2 Computational Biology and Bioinformatics Program, Duke University, Durham, North Carolina, United States of America; 3 Department of Molecular Genetics and Microbiology, Duke University, Durham, North Carolina; 4 Department of Medicine, Duke University, Durham, North Carolina, United States of America; 5 Department of Statistical Science, Duke University, Durham, North Carolina, United States of America; 6 Department of Computer Science, Duke University, Durham, North Carolina, United States of America; Lilly Singapore Centre for Drug Discovery, Singapore

## Abstract

Cancer is a heterogeneous disease often requiring a complexity of alterations to drive a normal cell to a malignancy and ultimately to a metastatic state. Certain genetic perturbations have been implicated for initiation and progression. However, to a great extent, underlying mechanisms often remain elusive. These genetic perturbations are most likely reflected by the altered expression of sets of genes or pathways, rather than individual genes, thus creating a need for models of deregulation of pathways to help provide an understanding of the mechanisms of tumorigenesis. We introduce an integrative hierarchical analysis of tumor progression that discovers which a priori defined pathways are relevant either throughout or in particular steps of progression. Pathway interaction networks are inferred for these relevant pathways over the steps in progression. This is followed by the refinement of the relevant pathways to those genes most differentially expressed in particular disease stages. The final analysis infers a gene interaction network for these refined pathways. We apply this approach to model progression in prostate cancer and melanoma, resulting in a deeper understanding of the mechanisms of tumorigenesis. Our analysis supports previous findings for the deregulation of several pathways involved in cell cycle control and proliferation in both cancer types. A novel finding of our analysis is a connection between ErbB4 and primary prostate cancer.

## Author Summary

Cancer is a complex disease with many subtypes that differ substantially with respect to their onset, progression, and response to treatment. Better understanding of the etiology and mechanism of cancer should help improve the diagnosis, prognosis, and treatment of cancer that will kill more than half a million Americans this year alone. Our study illustrates how integration of data over multiple stages and modeling tumorigenesis at the level of regulatory pathways or sets of genes provide robust and interpretable novel hypotheses concerning root genetic causes responsible for cancer initiation, progression, and invasion. Our modeling approach is one of the first approaches that combines multiple microarray datasets in a truly integrative framework that promotes the interpretability of important factors or pathways in one or more datasets. We apply this analysis of tumor progression to both prostate cancer and melanoma to provide information that can lead to the identification of novel biomarkers and give a basis for how genetic disruptions serve to alter actions in specific cell types.

## Introduction

In the past several decades, many genes have been discovered that govern important functions in the development of a variety of different cancers. However, biological insight from the list of genes is still limited and the underlying mechanisms that occur in the cell during tumorigenesis have not been well established. Numerous DNA microarray expression datasets have been collected to profile genetic changes throughout tumor progression [1–6]. Traditionally, gene expression profiling has been used to identify individual genes that become deregulated at distinct stages of tumorigenesis. Such analyses have shown that tumor cells have a great deal of heterogeneity as they progress through the stages of cancer development [7]. The multitude of differentially expressed genes can then be grouped together by shared biological function to uncover mechanistic alterations that may give rise to certain cancer states. This approach has resulted in the understanding of some of the genetic changes that occur during progression. However, single gene based methods do not always provide clear and accurate insight about the underlying biological processes governing tumor development since these processes involve sets of genes. Recently, gene set based methods have been developed to investigate phenotypic changes at the pathway level [8–11]. These methods provide an assessment of the enrichment of a group of genes defined a priori by some biological commonality for certain phenotypes. The main advantage of such methods over single gene based methods is that they begin with biological knowledge and therefore provide better functional or mechanistic insight into the cause of the phenotypic differences.

In this paper we provide an integrative hierarchical analysis of tumor progression which discovers a priori defined pathways that are relevant either throughout progression or in particular steps in progression. Pathway interaction networks are inferred for these relevant pathways over the steps in progression. This is followed by the refinement of the relevant pathways to those genes most differentially expressed over progression. The final analysis step is a gene interaction network inferred for these refined sets of genes. This analysis pipeline is applied to model progression in prostate cancer and melanoma.

The machine learning and statistical ideas used in the pipeline are regularized multi-task learning (RML) [12] and the ideas of learning gradients [13–16] and inverse regression [17,18]. The network inferences are based upon properties of discrete Gauss-Markov graphs [19].

## Results

We first validate the accuracy of the annotation of the pathway database used. We then describe in detail the application of our analysis to model progression of prostate cancer and melanoma.

### Validation of Gene Sets

The database of gene sets we use in this paper is the Molecular Signatures Database (MSigDB) [8]. This is a collection of curated gene sets from online pathway databases, publications in PubMed, and expert knowledge. Table S1 contains the MSigDB of gene sets used in the following analyses.

A key constraint in using a priori defined gene sets and pathways is the quality of the database of gene sets and the accuracy of the annotation. Since the enrichment of gene sets is fundamental to our models, we need to validate the accuracy of these gene sets. Two points will be addressed in our validation studies: the accuracy of gene sets annotated according to known perturbations and a comparison of gene sets defined by experimental studies versus gene sets defined by expert knowledge. An affirmative answer to the first point provides confidence in the annotation and interpretations made based on the annotations. A study of the second point highlights the importance of the context of the gene set and again provides information about interpreting results. An affirmative answer to this question allows for a uniform analysis over both types of gene sets.

The validation of gene sets requires the knowledge of which pathway gene sets are deregulated in which samples according to known biology. This requirement is satisfied by studies where a model system is genetically perturbed and a gene set is defined as genes that most differentially express under the perturbation. Expression studies where the pathways driving the phenotypic distinction are known also satisfy the above requirement. Due to the difficulty in finding data satisfying the above requirements for all the gene sets in our database [8], we focus on a few gene sets which we can validate: the P53 pathway, the hypoxia pathway, and the RAS pathway. The conclusion of our analysis will be that both experimental and expert defined gene sets seem to be accurately annotated and gene sets defined by expert knowledge may be applicable to more general conditions.

#### P53 pathway.

Our database of gene sets [8] contained 5 sets defining P53 pathways both from experimental studies and from expert sources. We first test whether these five gene sets will be significantly enriched in a dataset with a known P53 perturbation from the NCI-60 collection of cancer cell lines. This dataset includes 12 normal samples and 50 samples with a P53 mutation. Using GSEA, the normalized enrichment score (NES), *p*-value, and false discovery rate (FDR) for the 5 P53 pathway gene sets along with the other gene sets in the MSigDB were obtained. We found that the P53 pathway gene sets were all significantly enriched in the P53 wild type cells (results summarized in Table 1). These results give confidence that the 5 P53 signatures, although from different sources, are accurately representing the P53 pathway.

**Table 1:**
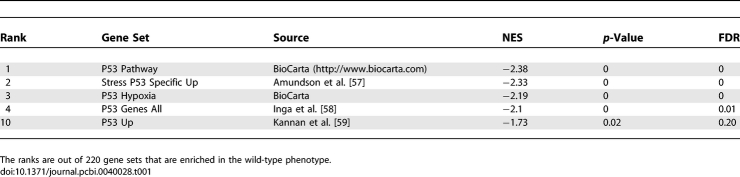
GSEA Results for the P53 Gene Sets in the Wild-Type/P53 Mutant Dataset

Further validation of the P53 pathway gene sets comes from an indirect comparison based on P53 as a marker for recurrence of breast cancer [20–22]. In [5], gene expression profiles were obtained for 60 individuals with hormone receptor-positive primary breast cancer treated with adjuvant tamoxifen monotherapy. Of these individuals, 32 experienced tumor recurrence. All of the P53 pathway gene sets except “P53 Up” differentiate the nonrecurrent from recurrent breast cancer phenotypes and rank highly based on their enrichment scores (see Table 2). The fact that “P53 Up” did not rank highly is not surprising as it is also the gene set with the worst enrichment in the NCI-60 dataset. The ability of the other four gene sets to differentiate P53 perturbations and breast cancer phenotypes provides confidence in using these gene sets.

**Table 2:**
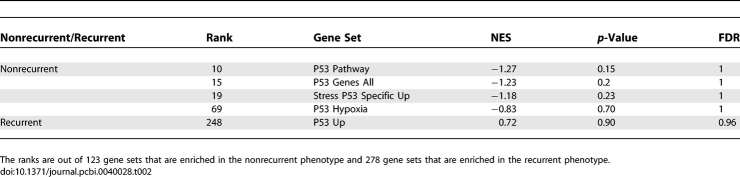
GSEA Results for the P53 Gene Sets in the Recurrent/Nonrecurrent Breast Cancer Dataset

#### Hypoxia pathways.

Our database of gene sets [8] contained 7 gene sets defining hypoxia pathways (see Table 3) both from experimental studies and from expert sources. Hypoxia occurs when a cell experiences an oxygen deficiency. The cell responds to the low oxygen environment by activating certain hypoxia pathways which induce numerous adaptive responses. Alternatively, cancer cells can genetically activate a hypoxia response in the setting of normal oxygen levels to activate new blood vessel formation and experience a growth advantage. Recently, much work has been done to better understand cellular responses to hypoxia conditions for therapeutic targeting of cancer cells which have the ability to adapt to these conditions and alter certain signaling pathways to increase survival.

**Table 3:**
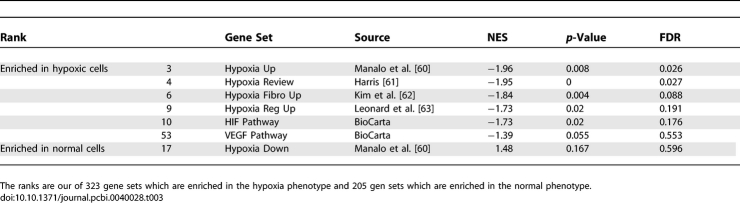
GSEA Results for the Hypoxia Gene Sets in the Hypoxia/Normal Dataset

The enrichment of these 7 gene sets were examined in an expression dataset which profiled 3 human astrocytes and 3 epithelial cells (HeLa cells) maintained under hypoxic conditions and 3 human astrocytes and 3 HeLa cells maintained under normal conditions [23]. GSEA was applied to this dataset to examine the enrichment of the gene sets in the database with respect to the 6 hypoxic and 6 normoxic cells. The enrichment scores of the 7 hypoxia gene sets rank highly out of the 528 gene sets tested from the MSigDB for their ability to discriminate hypoxic and normoxic cells in both cell lineages (astrocyte and epithelial cells) (see Table 3). This provides further support for using gene sets defined for the same biological process from a variety of sources.

#### RAS pathway.

The final example illustrates the importance of context in the pathway approach. The enrichment of three RAS pathways gene sets were examined under two conditions or datasets. The context of the first dataset is very specific, an animal model with mutated KRAS [24]. The second dataset is much more heterogeneous, non-small cell lung cancers [25]. The three gene sets correspond to two experimentally derived gene sets [24,26] and the RAS pathway from BioCarta (http://www.biocarta.com). The RAS signature from BioCarta is the most general with respect to context as it consists of genes thought to biochemically interact with RAS and proteins associated with RAS. The two experimentally defined gene sets are much more context specific. The first gene set corresponds to a pathway signature derived from infecting human primary mammary epithelial cell cultures with an adenovirus expressing activated HRAS [24]. The second gene set is a pathway signature derived from a mouse model with a KRAS mutation. In summary these two gene sets annotate HRAS and KRAS deregulation. Although the three sets all describe the RAS pathway, there are very few shared genes between the sets, emphasizing the importance of context when defining gene sets. The HRAS gene set and KRAS gene set share 8 genes and contain 237 and 58 unique genes respectively. The RAS gene set defined by BioCarta contains 23 genes, sharing only 1 gene with the HRAS gene set and having no genes in common with KRAS gene set.

The importance of the context in which a gene set was defined is demonstrated by testing the enrichment of the HRAS gene set on a dataset with a KRAS mutation. The HRAS gene set was split into the genes up-regulated and down-regulated in response to RAS activity. In [24], microarrays were collected from 31 cells with tumors caused by a KRAS mutation and 19 normal cells. Using GSEA, the NES, *p*-value, and FDR for the HRAS up-regulated and down-regulated gene sets were obtained. The oncogenic pathways of BCAT, E2F3, MYC, and SRC were also defined in [24] and were added to the analysis for enrichment comparisons. Results are shown in Table 4. The experimental HRAS gene set is narrowly defined for genes perturbed by HRAS activation and does not capture KRAS mutation specificity. On the other hand, the RAS gene set defined by BioCarta is appropriate to use regardless of the specific RAS mutation as it captures the interactions that the RAS protein can have under different conditions.

**Table 4:**
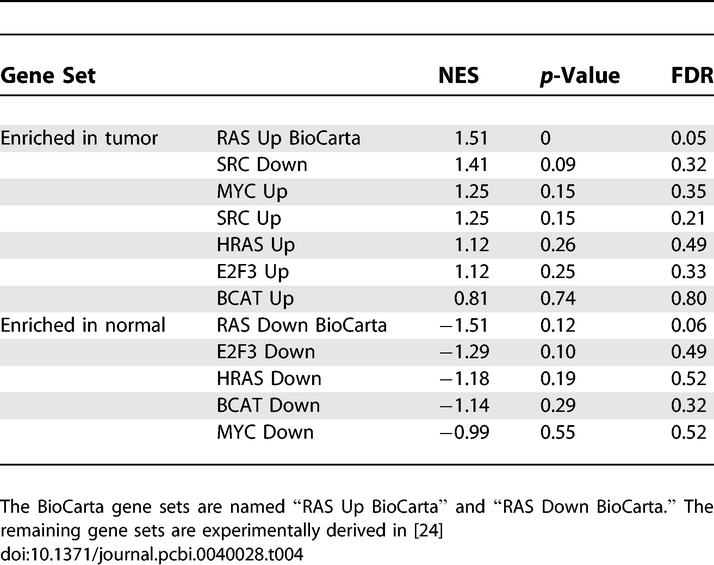
GSEA Results for the Oncogenic Gene Sets Defined [24] and BioCarta in the KRAS Mutant/Normal Dataset

The context of the RAS gene set does not have as much of an impact on datasets that indirectly involve RAS perturbations, even though the different RAS gene sets share a limited number of genes. Previous studies have shown that RAS activation is linked with lung adenocarcinomas [27,28]. The BioCarta RAS signature, the HRAS signature, and the KRAS signature were tested for their diagnostic capabilities in human lung cancer using a gene expression dataset with 45 adenocarcinoma lung cancer samples and 48 squamous lung cancer samples [25]. Using ASSESS [9], enrichment scores were calculated for each sample in the three RAS gene sets. The class prediction accuracy based on enrichment score was 69.9% for the HRAS pathway, 75.3% for the KRAS pathway, and 79.6% for the BioCarta RAS pathway.

These results give confidence for the robustness of ASSESS in allowing for the use of both literature and experimental based gene sets when the dataset contains a more general perturbation. However, if a dataset is built on a perturbation of a specific proto-oncogene, enrichment will not be seen in a gene set for a different proto-oncogene, even if these two proto-oncogenes belong to the same family. Therefore, knowing the context under which both the dataset and the gene set were built is important for interpreting results of enrichment analyses.

### Modeling Framework for Cancer Progression

We first state a summary of the framework we use to model tumor progression. A detailed description of the steps in the analysis and the methods from machine learning and statistics used is provided in the methods section.

The analysis can be divided into three objectives that fall into a natural hierarchical framework—an analysis at the pathway level to identify important pathways and build pathway networks, followed by a gene level analysis to refine relevant pathways and then infer a gene network for those relevant pathways. The first two objectives are at the pathway level. The first objective is to determine which pathways are most relevant to progression over both transitions, normal tissues to primary tumors (*n* ↦ *p*} and primary tumors to metastasizing tumors (*p* ↦ *m*}, and those relevant to one transition or the other. The second objective is to estimate the interdependence of pathways relevant to each step of tumor progression. This provides a pathway network for each step of tumor progression. The third objective, the refinement of relevant pathways, is at the gene level. The refinement procedure removes genes in relevant pathway gene sets that are not relevant to progression, resulting in a “refined” gene set. A gene network for each refined gene set can be inferred by estimating the interdependence between genes. In the following two subsections we apply this framework to prostate cancer and melanoma.

### Prostate Cancer Progression

The prostate cancer data [1] is a collection of cDNA microarray expression measurements from 22 samples of benign epithelium (b), 32 samples of primary prostate cancer (p), and 17 samples of metastatic prostate cancer (m). The progression is benign to prostate cancer (PCA) to metastasis, (*b* ↦ *p* ↦ *m*}. We will follow the framework outlined in the previous section for the analysis.

At each level of the analysis we use the six hallmarks of cancer [29] to organize the analysis results. These hallmarks, thought to be necessary for the development of cancer, were defined as self-sufficiency in growth signals, insensitivity to anti-growth signals, evasion of apoptosis, limitless replicative potential, sustained angiogenesis, and tissue invasion and metastasis. It was hypothesized that tumors must acquire alterations in each of these categories in order to evade the multitude of anticancer defense mechanisms in the cell. We use these hallmarks as categories to group gene sets found to be relevant in our analysis. The gene sets are assigned to the biological category most fitting, although there are cases where a gene set may fall into more than one category. In addition, pathway gene set dependence graphs inferred will illustrate the extent to which the hallmarks are interwoven. Pathways gene sets corresponding to all six hallmarks were found to be relevant.

#### Pathways relevant in prostate cancer progression.

**Figure 1:**
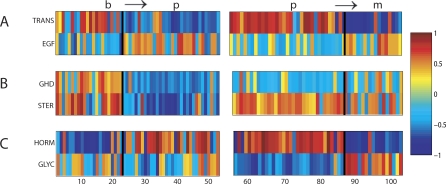
Pathways Relevant to Progression The top figure displays enrichment scores of the two gene sets with the smallest p-values for relevance throughout progression (*b* ↦ *p* ↦ *m*} based on RML. The middle figure displays two gene sets with the smallest p-values for relevance to the early transition (*b* ↦ *p*} based on RML. The bottom figure displays the two gene sets with the smallest p-values for relevance to the late transition (*p* ↦ *m*} based on RML. The enrichment scores are plotted from both transitions, (*b* ↦ *p*} and (*p* ↦ *m*}. Each column represents a sample. As such, the samples from the primary stage are plotted twice since they are used in the enrichment analysis of both transitions. For each transition, the samples within the primary stage are plotted in the same order. See Table 5 for full pathway names.

The first result of our analysis is a list of pathways and gene sets relevant to progression in prostate cancer (see Table S2). In addition, the analysis indicates in which stage of progression a pathway is relevant: both steps in progression (Figure 1A), the first transition (Figure 1B), or the second transition (Figure 1C). See Table 5 for the names and description of the gene sets in Figure 1.

**Table 5:**
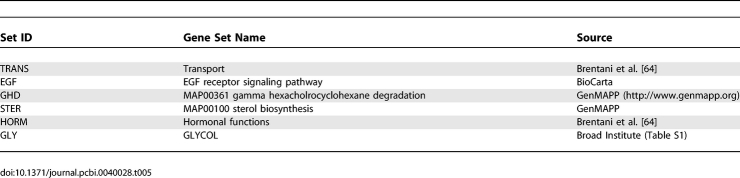
Gene Set Names for the Gene Set IDs in Figure 1

In the three cases, a *p*-value has been assigned to each of the 522 gene sets in the analysis. This *p*-value, calculated by random class label permutations (see methods section), assumes a null distribution with exchangeable labels. For each of the three stages in progression, we chose those gene sets with *p* ≤ 0.05 as relevant. This resulted in 29 gene sets relevant to overall progression, 17 gene sets relevant in early progression, and 22 gene sets relevant in late progression. These relevant gene sets will be discussed in the remainder of this section.

Pathway gene sets known to be involved in self-sufficiency in growth signals were found to be relevant over all stages of progression. In the list of significant gene sets relevant through both stage transitions several cell cycle gene sets are present. These gene sets correspond to general function required for the cell cycle to run normally. For instance, the cell cycle checkpoint gene set defined by Gene Ontology is found to be relevant. This gene set is comprised of genes involved in checkpoints for the spindle formation, DNA integrity, G2/M transition, G1/S transition, cell size, and meiotic recombination. The relevance of this gene set gives an idea of the general perturbations intrinsic to the tumor cell. However, a more detailed understanding of the mechanism for tumor progression and proliferation can be gained by studying relevant gene sets corresponding to particular pathways. Both the ErbB4 pathway and EGF receptor signaling pathway are found to be enriched in all stages of tumor progression. These two pathways are related. ErbB4 (or HER4) is a member of the EGF receptor family and is involved in growth signaling. Therefore, it is reasonable to hypothesize that one of the underlying mechanisms in self-sufficiency in growth signals may be the deregulation of the EGF cell surface receptor ErbB4, which results in the increased transduction of growth stimulatory signals into the cell. Further evidence of the importance of EGF signaling is seen with the ERK pathway. Like the EGF pathway, the ERK pathway is involved in RAS signaling and thereby increases proliferation and development [30]. The behavior of the Hormonal Function gene set is of great interest. The enrichment of this gene set decreased in the prostate cancer to metastatic transition. This trend has been documented in prostate cancer where during progression the tumors become androgen independent resulting in decreased androgen production in the cell [31].

Pathways corresponding to insensitivity to anti-growth signals were also found to be relevant in our analysis. Throughout progression, the PTEN pathway was shown to have decreasing enrichment and the PTDINS pathway was shown to be up-regulated. These two pathways are closely linked. PTDINS signals PTEN which is a tumor suppressor gene that is often mutated in prostate cancer. PTEN down-regulation, as well as PTDINS activation, leads to the activation of the AKT pathway and anti-apoptotic activity [31].

Pathways corresponding to the evasion of apoptosis were found to be relevant at all stages of prostate progression. The TNF and FAS Network gene set and the ROS gene set were found to be significant. FAS is a member of the TNF receptor family normally involved in cell death. The down-regulation of this pathway seen in late progression can be implicated with anti-apoptotic activity. ROS was shown to be down-regulated throughout progression. ROS generation in mitochondria activates caspase-3 and leads to a pro-apoptotic activation cycle [32]. The deregulation of ROS therefore may be an anti-apoptosis mechanism important in prostate cancer.

Although pathways corresponding to limitless growth were not as prominent as the first three hallmarks, we do see one of the classic markers of this characteristic, HTERT up-regulation. The length of telomeres indicate the number of cell generations elapsed. With each generation, telomeres lose 50–100 base pairs and when the telomeres get to a critical length, the cell undergoes senescence [33]. The gene set for the telomerase enzyme HTERT is shown to be up-regulated in late progression. HTERT over-expression maintains telomere length, thereby enabling unlimited replicative potential [34].

The analysis does not reveal specific angiogenesis gene sets relevant to progression. Previous research has found hypoxia signatures associated with poor prognosis of many cancer types including breast and ovarian, yet not prostate cancer [35]. The analysis does however indicate many metabolic pathways, giving evidence for the cell's need for additional nutrients as the cell grows and proliferates.

It has been previously documented that the capacity for invasion is a multi-step process and the process of acquiring the numerous needed perturbations often begins early in the disease process [36]. The results support this finding, indicating several pathways associated with invasion to be relevant early in progression. For example, the mCalpain pathway was found to be up-regulated in the benign to PCA transition. This pathway is associated with invasion through the regulation of integrin-mediated cell migration [37].

An interesting observation we find is that many gene sets involved in energy production rank highly in the analysis. There is a glycolysis pathway significant to the overall progression and two additional glycolysis pathways relevant to the late transition. In the early transition, the oxidative phosphorylation gene set is also up-regulated. The up-regulation of each of these gene sets describes the cell's continual need for energy as it undergoes additional replications and eventually invasion and metastasis.

#### Preliminary validation of pathways relevant in prostate cancer progression.

We carried out two analyses to validate our results from the above section. The first validation metric was the leave-one-out cross-validation error. The accuracy was 96.11%, giving confidence to the robustness of the RML model.

We next performed a preliminary validation of the pathway gene sets found to be relevant in our analysis by examining their enrichment in an independent prostate cancer dataset [38]. This dataset consisted of local and metastatic prostate cancer samples. The enrichment of the same 522 gene sets was computed using GSEA with respect to the local versus metastatic class labels. The validation metric was the overlap in the top 20 gene sets from the ranked list obtained from the NES from GSEA to the top 20 gene sets from the ranked list of gene sets found to be most relevant in the prostate cancer to metastatic transition. There were 8 gene sets in the overlap corresponding to a *p*-value of 9.2 × 10^−8^ using the hypergeometric distribution. Included in this overlap are several cell cycle control and energy production gene sets. This result gives confidence that the gene sets identified in our analysis are indeed significant for disease progression. We again see the utility of using gene sets instead of single genes as a similar analysis done with single genes resulted in no overlap.

#### Pathway dependency structure in prostate cancer progression.

Given inferences of pathways relevant to each step of tumor progression, the second objective is to understand how these pathways interact at specific stages of progression. The goal here is to infer a pathway interaction network for each stage of progression. This information will provide knowledge on the relationship between gene sets at a higher level—how certain pathways relate, interact, and influence each other with respect to the phenotype being studied, thereby creating a dependency structure of the pathways. The relationships are visualized by an undirected graphical model. We do not claim nor model causality in these graphical models, however we believe that causative relationships may be suggested that can be further experimentally tested.

We first look at three dependency relationships of interest. As previously discussed, the PTEN and PTDINS pathways are known to be closely associated. This is supported in the analysis by the dependency relationship between the two pathways. PTDINS is the 15th ranked pathway out of 522 based on the covariance with the PTEN pathway throughout progression. Likewise, the IGF1R and ERK pathways are known to be linked through their common association with the RAS pathway. We find support for their association as the ERK pathway ranks 9th based on the covariance with the IGF1R pathway during overall progression. Finally, PTDINS ranks 32nd based on the covariance with IFG1R, recapitulating the known relationship between these two pathways. These findings support the ability of our analysis to identify pathways that are closely associated.

**Figure 2:**
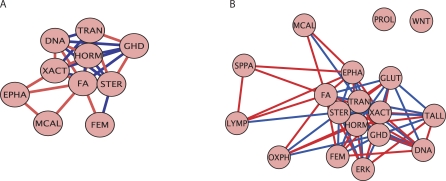
Pathway Association Graphs An association graph of (A) the top 10 gene sets in the (*b* ↦ *p*} transition and (B) the gene sets with *p* < 0.05 for the (*b* ↦ *p*} transition. The red lines indicate a positive dependence while the blue lines indicate a negative dependence. Distances between gene sets reflect the strength of dependencies. See Table S2 for full pathway names.

As previously discussed, *p*-values were calculated for each of the 522 gene sets in the analysis. We chose the ten gene sets with the lowest *p*-values in the benign to prostate cancer step in progression. The dependency structure of these gene sets are displayed in Figure 2A. Although these ten gene sets are all significant in differentiating the benign and PCA samples, the dependency structure shows that some may be more influential than others. For example, there is a group of 7 gene sets which cluster closely together, indicating that the deregulation of these genes sets play a key role in the phenotypic change from benign to PCA. We call these the core gene sets for the early step in progression. Gene sets with fewer connections are also important in phenotypic determination, however, the enrichment of these gene sets over all of the samples differ slightly from the core group. The assumption here is that one, or a few, of the gene sets in the core group are influencing the expression of the other gene sets, both in the core group and on the outside connections. The Sterol Biosynthesis and FA (Fanconi anemia) gene sets are both highly connected to the other gene sets. These are good candidates for pathways which are strongly influencing the expression pattern of other pathways.

Further information on gene set interactions can be gained by increasing the number of gene sets examined and displaying the dependence structure. For example those gene sets with *p* ≤ 0.05 are shown in Figure 2B. Most of the gene sets discussed previously were relevant throughout progression and therefore do not appear in this network of sets describing the early transition. The few gene sets discussed that are relevant in the early transition do indeed appear in the network, such as the ERK pathway and Hormonal Function gene set. The gene sets which clustered tightly in Figure 2A still cluster tightly in the middle of Figure 2B. However, several additional gene sets are shown to have strong dependencies with these gene sets as well. The additional gene sets are important in the phenotypic transition but have slightly different enrichment patterns as the top 10 gene sets. Aside from the Sterol Biosynthesis and FA gene sets, the Hormonal Functions gene set has the largest number of connections, indicating that it may also be governing expression patterns. It is further apparent that gene sets from each of the hallmarks of cancer interact with one another. Therefore, although each hallmark represents a unique characteristic, they are not separate entities but rather influence one another.

#### Refinement of pathways in prostate cancer progression.

The pathway level analysis provides us with inferences of which pathways are relevant over the two steps of prostate cancer progression as well as the interaction between pathways. The next level of analysis is the refinement at the gene level. The pathways found to be relevant in each of the steps of progression are refined using procedures described in the methods section. The HRAS/KRAS gene set validation example provides an argument for refinement. Pathways available are not always in the right context for a specific dataset of interest. The refinement procedure adapts the gene set to the context of the dataset. Adding this gene set to the database of gene sets increases the diversity of the collected gene sets. In addition, a gene network modeling the interdependence of the genes in the refined gene set is inferred.

The refinement procedure consists of culling the pathway gene set to a “refined” set corresponding to the genes in the set most relevant to the transition. We applied the refinement procedure to the top 3 pathway gene sets in Table S2 based on *p*-values in the PCA to metastatic transition as well as 2 pathway gene sets with varying ranks. The refined gene sets were added to our database of gene sets and are listed in Table S3.

After adding the refined gene sets to our gene set database, the analysis was repeated to examine whether the new gene sets better differentiate phenotype as compared to the original sets. In all the gene sets analyzed, the refined gene sets rank higher than the original as expected since the refined gene sets represent the subset of genes most strongly correlated with phenotype (see Table 6).

**Table 6:**
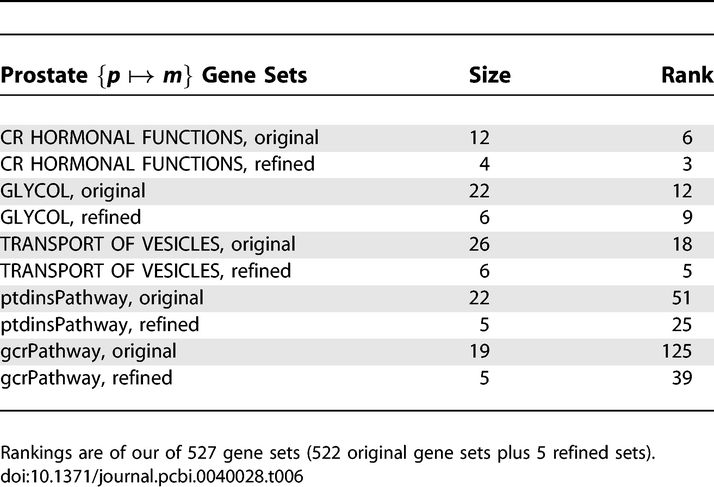
Rankings of Original and Refined Gene Sets for the PCA to Metastatic State Transition Based on RML

The refined gene sets were then analyzed on an independent prostate cancer dataset [38]. Using GSEA, we again see improvement in the rankings of the NES of each of the 5 refined gene sets over the original sets.

**Figure 3:**
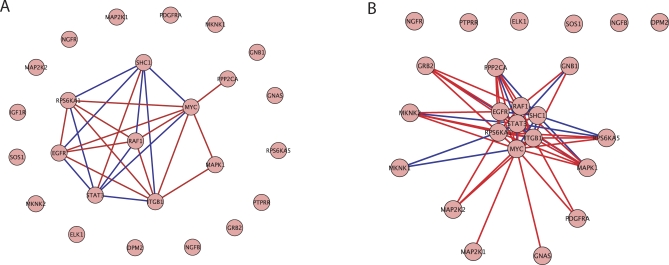
ERK Pathway Gene Association Graphs An association graph of the ERK pathway gene set with (A) a strong threshold on gene dependencies and (B) a weak threshold on gene dependencies. The red lines indicate a positive dependence while the blue lines indicate a negative dependence. Distances between genes reflect the strength of dependence between genes.

We also applied the refinement procedure to the ERK pathway gene set in the benign to PCA step in progression. Figure 3A and 3B displays all of the genes in the ERK pathway as defined in BioCarta and visualizes the pathway network. The difference between the plots is the threshold value below which dependencies are not displayed. The threshold is higher in Figure 3A than 3B so only the strongest interactions are displayed here. In Figure 3A and 3B there are 7 genes that all covary strongly with one another. These 7 genes are defined as the refined set. MYC is shown to have a strong relationship with the most genes in the gene set, being strongly dependent with respect to 8 genes. In addition to learning which genes are active in the pathway, Figure 3A displays numerous genes which are not relevant in this context. We conclude that the refined set of 7 genes, and in particular MYC, play an essential role in the ERK pathway deregulation seen in early prostate cancer progression.

#### Novel findings.

In addition to recapitulating previous findings for prostate cancer progression, new hypotheses have emerged out of our study. One interesting observation from the analysis is the relevance of ErbB4 in the early transition, suggesting the hypothesis that the ErbB4 pathway becomes deregulated in PCA individuals. The importance of EGF receptors have been documented in several cancer types, such as stomach, brain, and breast tumors [39,40]. HER2 has been implicated to be over-expressed in prostate cancer samples and thought perhaps to be related to the development of androgen independence [41]. However, currently little is known about a connection between ErbB4/HER4 and prostate cancer. Our analysis can motivate further research on defining the role of ErbB4/HER4 and prostate cancers.

Another interesting finding is the importance of the FA gene set in the early transition. The FA protein complex is known to associate with BRAC1 and BRCA2 and be involved in cell repair machinery [42,43]. Alterations in FA protein complex has been linked to several types of cancer including breast, brain, lung, and oral cancer [42,44].

### Melanoma Progression

We will further test our analysis on a melanoma tumor progression expression dataset. Genome-wide expression at different stages of melanoma development is available in [2]. Samples were categorized as normal (n), primary (p), or metastatic (m), with 4 individuals per group. The progression is normal to primary to metastasis, (*n* ↦ *p* ↦ *m*}. We follow the same hierarchical framework, analyzing relevant pathways, pathway networks, and finally gene networks.

#### Pathways relevant in melanoma progression.

**Figure 4:**
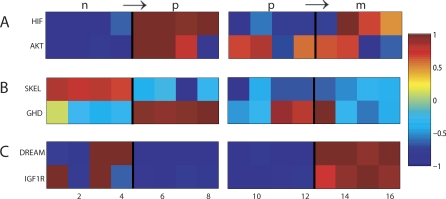
Enrichment Scores of the Two Highest Ranked Gene Sets from RML (A) (*n* ↦ *p* ↦ *m*}. (B) (*n* ↦ *p*}. (C) (*p* ↦ *m*}. See Figure 1 for further details and Table 7 for full pathway names.

The enrichment of gene sets with the lowest *p*-values for relevance throughout disease progression are displayed in Figure 4A, those most relevant in the transition from normal to primary are displayed in Figure 4B, and those for the transition from primary to metastatic are displayed in Figure 4C. See Table S4 for the full listing of pathways relevant with a *p* ≤ 0.05. The top two ranked gene sets for each transition are listed in Table 7.

**Table 7:**
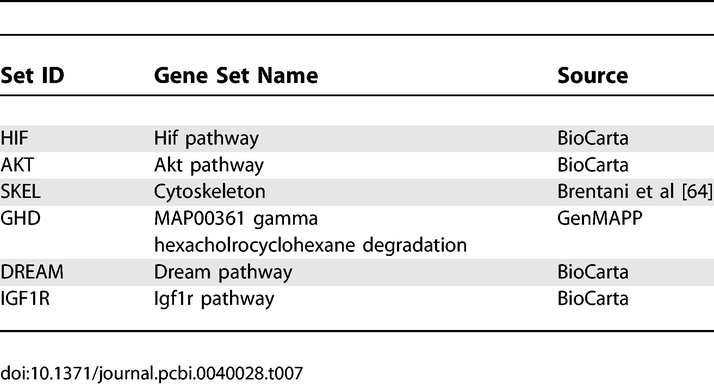
Gene Set Names for the Gene Set IDs in Figure 4

The results provide an understanding at the pathway level of the deregulation occurring in a developing melanoma cell to achieve the capacity for self-sufficiency of growth. Two well documented factors influencing self-sufficiency of growth are relevant to overall progression. The oncogene E2F is continuously over-expressed, increasing cell proliferation. Additionally, the AKT pathway is up-regulated which promotes cell survival and represses apoptosis. The AKT pathway is one of the effector pathways of RAS and can be activated by PTDINS over-expression, which we also see as relevant throughout melanoma progression. Our analysis suggests that the Sprouty pathway, which normally blocks EGF signaling [45] and prevents the activation of the RAS signaling pathway [46] is down-regulated.

The second hallmark of cancer, insensitivity to anti-growth signals, is also seen in melanoma. In the early transition, the CDK5 pathway is down-regulated. This alteration has been documented to lead to the activation of MEK1, a member of the MAP Kinase RAS effector pathway, and thereby to increased cell growth [30].

Several mechanisms for the escape from apoptosis are seen to be important in progression. Repression of apoptosis occurs through D4-GDI and IGF1R pathway over-expression and P53 and FA pathway under-expression. Decreased expression of the tumor suppressor P53 pathway throughout progression brings about unregulated continuation through the cell cycle, even when DNA damage is present [47]. The FA pathway has been implicated in several cancer types [42,44] but not yet reported in melanoma. Finally, the IGF1R pathway is found to be relevant to the late transition where the over-expression increases anti-apoptotic activity.

During melanoma progression, mechanisms involved in the defense against limitless replicative potential become deregulated. The HTERT gene set is shown to be up-regulated in early progression. HTERT over-expression maintains telomere length, thereby enabling unlimited replicative potential [34]. The fact that this alteration is seen in early disease development points to the importance of acquiring the capability for limitless growth in order to proceed into a neoplasia in addition to deregulation in other growth mechanisms.

Two important gene sets describing angiogenesis are seen during melanoma progression. The HIF pathway gene set shows over-expression throughout progression, indicating the neoplasia's continual increasing requirement for oxygen and nutrients. HIF-1 is activated during hypoxia leading to the induction of a network of genes involved in angiogenesis and glucose metabolism [48]. We again see the need for an increase in oxygen early in the disease by the Angiogenesis gene set that is found to be up-regulated at the early transition.

The final hallmark of cancer, invasion and metastasis, is acquired in melanoma progression. Cells gain the capacity for metastasis by alterations in proteins involved in cell-to-cell adhesion and cell-to-environment interactions [49]. We see proteins of this class to be altered through the deregulation of the Cytoskeleton gene set. The MTA3 pathway is down-regulated in the early transition. Decreased expression of MTA3 has been associated with ER-negative breast tumors. This down-regulation leads to the halt of E-cadherin production. E-cadherin is involved in coupling adjacent cells and transmitting anti-growth signals and is a widely observed alteration in cancers with invasion capacities [50].

#### Preliminary validation of pathways relevant in melanoma progression.

We carried out two analyses to validate our results from the above section. First, classification was applied in the leave-one-out setting (see methods). There were no errors in class prediction, giving confidence to the robustness of the RML model.

The second analysis involved an independent melanoma progression dataset [51]. This dataset consists of 9 samples of benign nevis, 6 samples of primary melanoma, and 19 samples of metastatic melanoma. The regression analysis was rerun on this dataset and several of the significant pathways from the first analysis were again found to be significant. In both analyses we found the down-regulation of the P53 pathway gene set throughout progression and the over-expression of the D4-GDI pathway and the HTERT gene sets at various stages in progression. The overlap of relevant gene sets from the two datasets [2,51] however was not found to be significant (*p* = 0.28). This may be attributed to the small sample size in the first dataset [2]. To further explore this issue, we randomly split the second dataset [51]. The regression analysis was run on the two resulting datasets independently. For each, we obtained a list of gene sets with *p* ≤ 0.05 for overall progression. Using the hypergeometric distribution, the overlap of the two lists was found to be significant, *p* = 0.018. This result gives confidence against overfitting and that the gene sets identified in the analysis are indeed significant.

#### Pathway dependency structure in melanoma progression.

**Figure 5:**
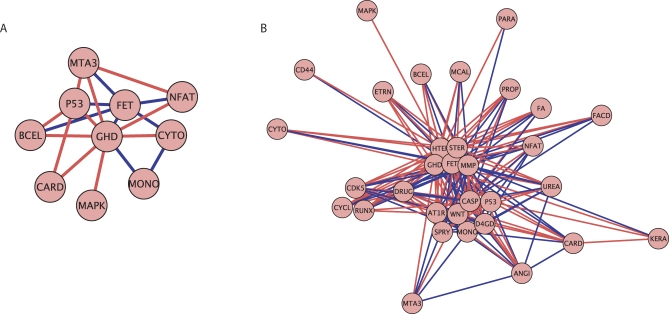
Pathway Association Graphs An association graph of (A) the top 10 gene sets in the (*n* ↦ *p*} transition and (B) the gene sets with *p* < 0.05 for the (*n* ↦ *p*} transition. The red lines indicate a positive dependence while the blue lines indicate a negative dependence. Distances between gene sets reflect the strength of dependencies. See Table S4 for full pathway names.

The dependency structure of the 10 pathways with the lowest *p*-values in the normal to primary transition is shown in Figure 5A. Although we do not have direct validation about the level of interaction of these pathways, we have confidence they are all significant in the (*b* ↦ *p*} transition from the RML analysis and their strong resulting *p*-values. We gain further confidence in their dependency structure through documented findings of similar interactions. As in the prostate cancer progression example, the Sterol Biosynthesis gene set is highly connected. Tumor cells have long been documented to have deficiencies in sterol synthesis. One component of the Sterol Biosynthesis gene set is the mevalonate pathway, which is a key intermediate in sterol production. In [52], it was found that tumor cells cannot synthesize mevalonate, but can obtain it from the host and then continue along in the sterol pathway. The dependency structure shows that the Sterol Biosynthesis gene set has a strong positive interdependence with the Fatty Acid Synthesis, Cyanoamino Acid Metabolism, and Gamma Hexachlorocyclohexane Degradation gene sets. The interdependence of these three gene sets with the Sterol Biosynthesis gene set rank 14th, 19th, and 3rd, respectively, out of the 523 pathways in the database. All of these gene sets may be closely tied to the inability of a tumor to synthesize certain metabolites and its increasing need for these metabolites as it grows and develops. Figure 5B displays the gene set dependency network for gene sets with *p* ≤******0.05. The top original top 10 gene sets cluster tightly, recapitulating their close relationships.

## Discussion

The major innovation presented in this paper is the use of gene sets in modeling tumor progression rather than single genes. Previous research in tumor progression has studied single genes that can be used as markers of certain stages [2,3]. While it is important to identify these individual genes, a broader understanding of the biological processes occurring during progression has been missing. Recent research in prostate cancer progression has taken a step closer to gaining insight into biologically related gene sets implicated throughout disease using the Molecular Concept Map [1]. This method identifies cellular functions that may be relevant based on common characteristics in the individual genes found to be differentially expressed. An analysis of breast and colorectal cancer was performed by [53], grouping genes using similar gene set databases, such as Gene Ontology and the KEGG Pathway Database. Unlike these analyses, we start in the space of gene sets rather than individual genes. We have introduced a novel analysis pipeline that discovers a priori defined gene sets relevant at different stages of the disease. In addition, an interaction network of these relevant gene sets is inferred. This is followed by refinement of the relevant pathways and gene sets to include only the genes most relevant to progression. A gene interaction network is inferred for these refined gene sets. This approach provides a more accurate and descriptive understanding of pathway deregulation by identifying specific pathway gene sets whose expression becomes altered along with phenotypic changes.

Since the method requires accurately defined gene sets, we first sought to validate the gene sets used in the analyses. P53, hypoxia, and RAS pathways have been defined by multiple sources, both in literature and experimental settings. For each, we took data with a specific perturbation for that pathway and calculated pathway enrichment. Results suggested that the gene sets are appropriate for use with careful attention to context. Context of both the dataset and gene set is important in enrichment analyses. We find this in the HRAS/KRAS example. Gene sets defined on one dataset may not be applicable for enrichment analyses when the context of the dataset changes. As such, careful attention should be paid for application of gene sets defined on one data set to a different context.

Our results largely agreed with findings in previous studies but also provide some novel biological insights into tumor progression. We discovered gene sets which become deregulated at certain stages and throughout progression. Common themes in the results presented in this paper and in [1] include an increased activity in the cell cycle, an increase of energy requirements, and an initial increase followed by a decrease in hormonal levels. The technique applied in [1] gave concepts which were relevant at certain stages of prostate cancer progression such as the concept of “cell cycle,” shown to have increased activity throughout progression. Our analysis takes this result a step further by discovering the specific pathways responsible for the increased activity, for example the ERK pathway in early progression. This provides better mechanistic insight into the process of proliferation during tumorigenesis.

In [2], individual genes were identified that were specific to create genetic profiles for different stages of melanoma. Although it is important to identify such markers that can differentiate stages, they do not provide an understanding of the underlying mechanisms that drive progression. [2] finds that genes involved in cell cycle regulation and proliferation are of utmost importance during melanoma development. Our method discovers several mechanisms underlying cell cycle regulation and proliferation that become deregulated such as the AKT and P53 pathways.

Studies have also been performed using comparative genomic hybridization (CGH) to associate DNA copy number aberrations with genetic progression [54,55]. We do not present such an analysis at this time; however, our analysis can be applied in this setting by using chromosomal regions as gene sets.

By transforming expression datasets into the space of enrichment scores of gene sets, we have extended previous research to gain insight into disease processes at the pathway level. We are able to study simultaneously the progression over multiple steps in tumor progression and provide pathway interaction networks of pathways relevant to these multiple steps.

## Methods

We first present an overview of the steps in the analysis, summarized in the data analysis algorithm below. We then proceed to explain in detail the algorithms and statistical models involved in each step of the analysis pipeline.

### Analysis pipeline.

The same data analysis algorithm or pipeline was followed for both the prostate cancer and melanoma examples.

The following are the analysis steps:

1. Stratify data: The expression data is stratified into *T* datasets corresponding to stages of progression. For example, if the progression is (*b* ↦ *p* ↦ *m*} then there are two steps, *T* = 2. The first dataset *D_1_* consists of samples of class *b* and *p* and the second dataset *D_2_* consists of samples of class *p* and *m*;

2. Map to summary statistics: The stratified data *D_t_* is mapped into a representation with respect to sets of genes or pathways, Γ, defined a priori. Pathways in this setting are genes putatively thought to co-express. Given the stratified data *D_t_* and a pathway database Γ, the summary statistic provides a measurement of the enrichment of each sample in *D_t_* with respect to each pathway in the database. If the dataset *D_t_* has *p* genes and *n* samples and there are *m* pathways in the database then the result of the summary statistic is a new dataset, *S_t_*, of the enrichment of the *m* pathways over the *n* samples.

3. Find pathways relevant to progression: The RML algorithm was applied to the mapped data 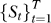 . The output of the RML algorithm are a set of vectors 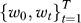 where the elements of *w*_0_ correspond to the relevance of a pathway over all stages of progression and the elements of *w_t_* correspond to the relevance of a pathway with respect to the *t*-th step in progression. A permutation procedure was performed to obtain *p*-values for each gene set in respect to each step in progression;

4. Leave-one-out cross-validation: Given data set 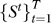 of enrichment scores, apply RML to training data in a leave-one-out setting. This results in an unbiased estimate of the error rate on the prediction of class labels.

5. Construct pathway association graphs: For the pathways found to be relevant over the *T* stages of progression construct a pathway association graph 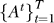 . Each *ij*-th element of *A^t^* indicates the dependence between pathways *i* and *j* conditioned on all other pathways and the relevance in modeling transition *t*.

6. Refine relevant gene sets: The pathways *G_t_* found to be relevant for the *t*-th step in progression are refined since not all genes in the pathway are relevant in modeling transition *t*. This results in a set of refined gene sets Γ*^R^* and *g* = | Γ*^R^* | is the number of refined gene sets.

7. Construct gene association graphs for refined pathways: For each refined gene set in Γ^R^ = 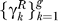 construct a gene association graph *Ã^k^* where the *ij*-th element of *Ã^k^* indicates the dependence between genes *i* and *j* conditioned on all other genes *γ_k_* and the relevance in modeling transition *t*.

### Stratification and mapping.

The first two steps in the analysis pipeline are stratifying the data and mapping the data into a representation based on pathways. The data can be represented as a set of pairs *D* = 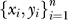 with *x_i_* ∈ *^p^* the expression over *p* genes and *y_i_* is the stage of the patient. Assume that there are three stages *y* ∈ (*b*, *p*, *m*} with *n_1_, n_2_, n_3_* samples in each stage and the progression is (*b* ↦ *p* ↦ *m*}. There are two steps in this progression, *T = 2*.

We first stratify the data with respect to these two steps. The first dataset *D*_1_ = 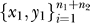 consists of the *n_1_* samples corresponding to stage *b* followed by the *n_2_* samples corresponding to stage *p* with the label of the first *n_1_* samples labeled as 0 (less serious) and the remaining *n_2_* labeled as 1 (more serious). The second dataset *D_2_* is constructed similarly with samples corresponding to stages *p* and *m*. The first *n_2_* samples are labeled as 0 (less serious) and the remaining *n_3_* labeled as 1 (more serious) in this dataset.

Each dataset *D_t_* is then mapped into a representation with respect to sets of genes or pathways. This is done using the pathway annotation tool ASSESS [9] that assays pathway variation in individuals. Given phenotypic or label data *Y_n_ =* (*y_1_,…, y_n_*}, expression data *X_n_ =* (*x_1_,…, x_n_*} and gene sets Γ = (*γ*_1_,…, *γ_m_*} defined a priori ASSESS [9] provides the summary statistic *S_n_* = *S*(*X_n_*, *Y_n_*, Γ}. The summary statistic *S_n_* is a matrix with *n* columns corresponding to samples and *m* rows corresponding to gene sets with each element S*_ij_* as the enrichment of gene expression differences in the *j*-th sample with respect to phenotype for genes in the *i*-th gene set. The application of ASSESS to the stratified datasets *D_1_*, *D_2_* results in two datasets *S_1_, S_2_*. The gene sets used in our analysis were those annotated in the MSigDB [8].

### Finding pathways relevant to progression.

The central statistical idea used in finding pathways relevant to progression is called multi-task learning [12] in the machine learning literature and hierarchical modeling with mixed effects in the statistics literature. We restrict ourselves in this paper to linear models and classification. The basic idea is that we have *T* classification problems in our case assigning a sample *x_i_* to labels 0 (less serious) or 1 (more serious). We assume that the classification tasks are related so the conditional distributions of the phenotype given the summary statistics *μ_t_*(*Y* | *S*) are also related. The tasks in our case are the different steps in tumor progression and the data over all tasks is *S = S_1_,...,S_T_* where *S_j_* = ((*y_1j_*, *s_1j_*),...,(*y_nj_*, *S_nj_*)} and *n_j_* is the number of samples in the *j*-th task. We assume the generalized linear model

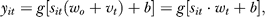
where *g* is a link function which for the SVM case is (*y_it_* − *s_it_* · *w_t_* − *b*)_+_, *w_t_ = w_0_ + v_t_*, *y_it_* is the *i*-th sample in task *t*, *s_it_* are the summary statistics of the *i*-th sample in task *t*, *w_0_* is the baseline term over all tasks, *v_t_* are the task specific corrections, and *b* is an offset. The vectors *w_t_* correspond to the linear model for each task.

We used the RML framework developed in multitask to estimate the model parameters *w*_0_, *v_t_*, and *b*

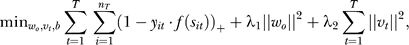
where (*u*)_+_ = min(*u*, 0) is the hinge loss, *f*(*s_it_*) *= s_it_* ·(*w_o_* + *v_t_*) + *b*, *λ*_1_ and *λ*_2_ are positive regularization parameters that trade-off between fitting the data and the smoothness or robustness of the estimates. In this paper we set *λ*_1_ = 1 and *λ*_2_ = 2,000, as to not over assume dependency between tasks.

Given the vectors *w_t_* we select gene sets corresponding to coordinates of the vectors with *p* ≤ 0.05 to find pathways relevant to the *t*-th step in progression, see [56] for details.

### Permutation procedure.

RML was repeated with 1,000 class label permutations to obtain a null distribution of each vector: 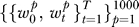 . The *p*-value for each gene set in *w_t_* was obtained by finding the percentile of *w_t_* in 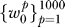 . Gene sets relevant to the *t*-th step in progression, *G_t_*, are those corresponding to elements of *w_t_* with *p* ≤ 0.05.

### Classification in the leave-one-out setting.

We applied the leave-one-out procedure for classification. The dataset 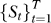 is split into *s_i_* (the *i*-th data sample) and *S* ^\*i*^ (the data without the *i*-th sample). RML is applied to the training set, *S* ^\i^ to build a classifier based on 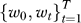 which is applied to *s_i_* to obtain a prediction 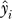 . Prediction accuracy is computed by applying the leave-one-out procedure to all samples in the dataset.

### Association graphs and refinement of gene sets.

The central statistical idea used in constructing association graphs as well as refining gene sets is learning gradients [13–16] and inverse regression [17,18]. These ideas apply to linear and nonlinear models but we restrict ourselves to the linear setting since we only use linear models in this paper. We first formulate the statistical ideas and then describe how this is applied in our application.

*Learning gradients, inverse regression, and graphical models:* The idea of inverse regression [17,18] is given the explanatory or input variables *X* and the output or response variable *Y* to study *X|Y* and more specifically Ω*_X_*_|*Y*_ = cov(*X* | *Y*):

1. The *i*-th diagonal element of this covariance matrix is a measure of the relevance of the *i*-th variable with respect to changes in the label or output variable;

2. The *j*-th off-diagonal element is a measure of the covariation between variable *i* and *j* with respect to changes in the label.

Estimating the inverse regression can be technically problematic if the covariance matrix is degenerate.

The idea of learning gradients addresses this technical problem. Given a regression or classification function *f* its gradient is

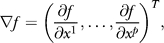
and the gradient outer product matrix (GOP) Γ is defined by its elements Γ*_ij_* = 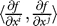 . In [16] the following relation between the GOP matrix and the covariance of the inverse regression Ω*_X_*_|*Y*_ was derived for linear functions

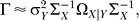
where Σ*_X_* = cov(*X*) for the inputs, and 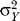 = var (*Y*) for the outputs. This states that Γ and Ω*_X_*_|*Y*_ are equivalent modulo a scale parameter (the variance of the output variable) and a rotation (the precision matrix of the input variables). In [14] an efficient algorithm to estimate the gradient and GOP matrix, Γ̃ given data for the classification setting was developed. We use this method in this paper.

The estimate of the GOP matrix Γ̃ is the covariance matrix of a multivariate Gaussian random vector by construction. The inverse of this matrix *J* = Γ̃^−1^ is by the theory of Gauss-Markov random fields [19] the conditional independence matrix (the pseudo-inverse is used when Γ̃ is degenerate),

*J_ij_* = dependence between variables *i* and *j* | all other input variables and the output variable.

*Construction of pathway association graph:* Given the list *G_t_* of pathways relevant to the *t*-th step in progression and the enrichment summary statistic dataset *S_t_*, this dataset is reduced by removing the enrichment scores of all pathways not in *G_t_*. The GOP estimate, Γ̃*_t_* , given this data is computed. This matrix is *d* × *d* where *d* is the number of relevant pathways in *G_t_*. The pseudo-inverse of this matrix *A_t_* = 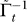 is the pathway association graph for the *t*-th progression step.

*Gene set refinement and gene association graph:* Not all genes in the relevant gene sets, *G_t_*, are differentially expressed between the two stages of progression in step *t*. We reduce or refine each of the pathway gene sets to those genes most relevant in progression step *t*.

The following procedure is iterated for each of the relevant gene set in *G_t_*. Given the *k*-th pathway gene set the stratified data *D_t_* is reduced to the genes in this gene set. The GOP estimate, Γ̃*^t^*, given this data is computed. This matrix is *d* × *d* where *d* is the number of genes in the *k*-th pathway in *G_t_*. Genes corresponding to large diagonal elements of the GOP matrix, 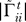
_ >_ *τ* , are those most relevant to the prediction and are the refined set *R*. In this paper, the threshold τ is selected such that we obtain a specific number of genes.

The GOP estimate, Γ̃*^t^*, is then reduced to only the genes in the refined gene set *R*. The pseudo-inverse of this matrix is the gene association matrix 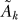 that provides the dependence of the refined gene set.

### Data analysis algorithm.

**Input**: data *D*, thresholds (*τ*_1_, *τ*_2_), gene sets Γ, RML algorithm *M*, graph algorithm *A*, refinement algorithm *R*.

**Return**: relevant gene sets 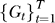 , pathway association graphs 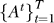 , refined gene sets Γ*^R^*, gene association graphs 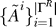


*T* = number of steps in progression;

**for t ← to** *T* **do**

/ / Stratify data by taking subset relevant in step *t*

*D_t_* ← *D*;

/ / Map *D_t_* to enrichment summary statistics over gene sets

*S_t_* = *S*(*D_t_*, Γ);


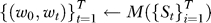
 ; / / Apply RML to the summary statistics

/ / Select relevant gene sets over stages of progression

**for t ←**1**to** *T* **do**

*G_t_* = Ø; Initialize relevant gene sets for stage *t*

 **for all** elements of *w_t_* **do if** |*w_ti_*|> *τ*_1_ **then** add gene set *i* to *G_t_*;

/ / Construct pathway association graphs

**for t** ← 1 **to** *T* **do** *A^t^* ← *A*(*G_t_*,*D_t_*)**;**

**/ /** Refine relevant gene sets

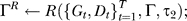



/ / Construct gene association graphs for refined gene sets

**for** *i* = 1 **to** |Γ*^R^*| **do** 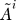 ← *A*(*D_t_*, Γ*^R^*);

## Supporting Information

Table S1 Database of Gene Sets (125 KB TXT)

Table S2 Gene Sets Significant in Prostate Cancer Progression (117 KB XLS)

Table S3 Refined Gene Sets from the Prostate Cancer Analysis (0 KB TXT)

Table S4 Gene Sets Significant in Melanoma Progression (99 KB XLS)
